# Enhanced switching and familial susceptibility for psychosis

**DOI:** 10.1002/brb3.988

**Published:** 2018-04-25

**Authors:** Fred W. Sabb, Gerhard Hellemann, Nicholas B. Allen, Carrie E. Bearden

**Affiliations:** ^1^ Lewis Center for Neuroimaging University of Oregon Eugene Oregon; ^2^ Semel Institute for Neuroscience and Human Behavior UCLA Los Angeles California; ^3^ Department of Psychology University of Oregon Eugene Oregon; ^4^ Brain Research Institute UCLA Los Angeles California

**Keywords:** cognitive control, Heritability, Internet, phenotype, resilience, set shifting, vulnerability

## Abstract

**Introduction:**

Working Memory and Task‐Switching are essential components of cognitive control, which underlies many symptoms evident across multiple neuropsychiatric disorders, including psychotic and mood disorders. Vulnerability to these disorders has a substantial genetic component, suggesting that clinically unaffected first‐degree relatives may carry some vulnerability‐related traits. Converging evidence from animal and human studies demonstrates that dopamine transmission, striatal and frontal brain regions, and attention and switching behaviors are essential components of a multilevel circuit involved in salience, and disruptions in that circuit may lead to features of psychosis. Yet, it is possible that unaffected relatives may also possess characteristics that protect against development of illness. We hypothesized that reduced switch cost in a cued task‐switching task, may be a behavioral expression of this “resilience” phenotype that will be observable in unaffected relatives.

**Methods:**

We tested a large community sample (*n* = 536) via the web, to assess different subcomponents of cognitive control, including task‐switching and working memory, as well as risk‐taking, among individuals who report having an affected relative with a psychotic or mood disorder.

**Results:**

Healthy individuals with suspected genetic risk due to a self‐reported familial history of a psychotic disorder demonstrated better task‐switching performance compared to healthy people without a psychiatrically ill relative and those with a relative with a mood disorder. This result was specific to illness status and task domain, in that individuals with a personal history of depression or anxiety did not show improved task‐switching performance, and this improvement was selective to task‐switching and not seen in other putative cognitive control domains (working memory or risk taking).

**Conclusions:**

Although this study has limitations and independent replication is needed, these preliminary findings suggest a potential avenue for understanding susceptibility to these disorders by highlighting possible protective as well as vulnerability‐related aspects of risk phenotypes.

## 1 INTRODUCTION

Cognitive control can be defined as the ability to flexibly execute goal‐directed behavior with sensitivity to both changing external exigencies and internal goals; this concept encompasses multiple processes including the maintenance of plans for action, the inhibition of prepotent responses, and both passive and active updating of that action plan (Miller & Cohen, 2001). While the latent processes and task dimensions associated with this overarching construct remain actively studied, working memory is consistently named as a primary component of cognitive control in the literature (Sabb et al., 2008). The ability to switch between two competing tasks (task‐switching) is also frequently considered a subcomponent of cognitive control in the literature (Sabb et al., 2008). There is significant overlap in the paradigms and putative neurobiological underpinnings relevant to all three latent constructs (Sanislow, Pine, Quinn, & Garvey, 2010), indicating a need for empirical data to help determine the unique and overlapping features among components of cognitive control, at behavioral and neurobiological levels.

A wealth of evidence suggests that cognitive control is impaired in a number of highly heritable neuropsychiatric syndromes ranging from attention deficit disorder to mood disorders like depression and bipolar disorder (Altshuler et al., 2004; Barch & Sheffield, 2014; Green, Cahill, & Malhi, 2007) to schizophrenia (Glahn et al., 2003; Walshaw, Alloy, & Sabb, 2010; Willcutt, Doyle, Nigg, Faraone, & Pennington, 2005), and that those at genetic risk for these disorders also frequently show at least mild impairments consistent with a cognitive endophenotype. Working memory, in particular, is considered a hallmark deficit in patients with schizophrenia (Glahn et al., 2003), and it is established that unaffected siblings of patients with schizophrenia also demonstrate similar but milder cognitive impairments (Macdonald, Pogue‐geile, Johnson, & Carter, 2003; Snitz, Macdonald, & Carter, 2006). The contribution of “task‐switching” is not as well studied. While deficits in complex tasks that involve switching, such as the Wisconsin Card Sorting Test (WCST), have also been observed in both patients with schizophrenia (Egan et al., 2001) and their unaffected relatives (Snitz et al., 2006), the contribution of the switching construct itself to these deficits has been challenged. Neuropsychological tests typically used in clinical populations, such as the WCST, likely draw significantly on working memory and thus may conflate impairments in these putatively separate processes (Meiran, Chorev, & Sapir, 2000; Miyake, Emerson, Padilla, & Ahn, 2004). Several studies have reported that tasks that tap switching might be relatively spared in schizophrenia (Greenzang, Manoach, Go, & Barton, 2007; Li, 2004; Manoach et al., 2002; Meiran, Levine, Meiran, & Henik, 2000; Wylie, Clark, Butler, & Javitt, 2010) and thus the association of switching abnormalities in schizophrenia, and risk for schizophrenia, remains an open question. Similarly, in depression, studies examining switching using classic clinical tasks such as WCST, show consistent impairments in depression (Austin, Mitchell, & Goodwin, 2001). Recent work using paradigms from the cognitive neuroscience literature also shows task‐switching performance is reduced in young adults with high familial risk for mood disorders (Papmeyer et al., 2015), and those that developed major depression showed a decrease in switching ability at follow‐up (Papmeyer et al., 2015). More recent evidence, however, posits that switching deficits in depression might be only in those with ruminating symptoms (Whitmer & Gotlib, 2012).

While a substantial body of research has examined links between impairments in cognitive control and genetic risk, specifically targeting “endophenotypes” that by their definition should show performance decrements in clinically unaffected siblings (Gottesman & Gould, 2003), few studies have examined the cognitive features of clinically unaffected relatives that may suggest favorable adaptation or putative protective factors from illness. Crow and others (Brüne, 2004; Crow, 1997, 2000) have argued that there must be beneficial aspects to risk phenotypes that have allowed these genes to survive natural selection. Unaffected relatives therefore should carry some expression of “resilience.” If subcomponents of cognitive control including working memory reliably impair functioning in both affected individuals and those at genetic risk, switching may be a phenotype that confers benefits or enhanced ability above healthy participants without familial risk. This benefit, however, likely depends on environmental contingencies, as enhanced ability to execute switching between tasks could be seen as “creative” (appropriately flexible) or “loose” (excessively labile or unstable) depending on the context [e.g., (Ramey & Chrysikou, 2014)]. By examining healthy individuals with familial risk, we may uncover this enhanced switching phenotype. Prior work by multiple groups emphasizes a dimension of cognitive and neural systems function with poles reflecting cognitive flexibility and stability, and specifically the roles played by dopamine transmission in these processes (Bilder, Volavka, Lachman, & Grace, 2004; Durstewitz, Kelc, & Güntürkün, 1999; Miller & Cohen, 2001; O'Reilly, 2006; Robbins & Arnsten, 2009). If a balance between stability and flexibility is important for typical cognitive processing, we might hypothesize that when genetic risk pushes people toward the tails of that distribution, there would be a point before they succumb to frank disease where we might see a unique phenotype. Thus, if healthy individuals with suspected genetic risk due to a family history of psychosis do not have significant clinical deficits despite some shared neurobiological vulnerability, then it would be valuable to determine not only the risk factors that show vulnerability, but the potential expression of behavior that was “protective” or allowed them to escape their increased risk. Given the strong evidence of impairment in working memory, the lack of strong evidence for a specific switching deficit, and neurobiological evidence related to salience, we posited switching might be that phenotype.

We hypothesized that healthy individuals with suspected genetic risk due to a self‐reported familial history of psychosis would show more efficient switching, as measured by a reduced cost of switching between tasks, relative to healthy individuals with no family history of a psychosis spectrum diagnosis. We were also able to examine the specificity of this hypothesis by examining those with a family history of a mood disorder without psychosis. If these individuals were able to overcome their familial risk, and the likely performance decrement on many indicators of working memory/stability frequently seen across studies of relatives, a potential candidate behavior might be increased behavioral flexibility.

We further hypothesized this would be a specific benefit seen only in tasks that putatively tap flexibility, like switching. In order to determine the specificity of our hypothesis alongside other putative indicators of cognitive control, especially given the overlap of paradigms, and the fluidity of the construct, we attempted to separate component cognitive processes mechanistically using well‐known tasks related to stability such as working memory as well as putative impulse control/risk‐taking measures like the BART. While the BART is not a traditional cognitive control task, it has been examined in relationship to cognitive control (Bogg, Fukunaga, Finn, & Brown, 2012; Brown & Braver, 2008; Knoch et al., 2006; Kohno, Morales, Ghahremani, Hellemann, & London, 2014; Mills, Goddings, Clasen, Giedd, & Blakemore, 2014; Rao, Korczykowski, Pluta, Hoang, & Detre, 2008), although not generally associated with a dimension of cognitive stability/flexibility and was chosen to assess the contribution of inhibitory processes. We also hypothesized this effect would only be seen in those with no expression of their familial risk, suggesting “resilience” or compensatory mechanisms.

We used a web‐based platform to test a large community cohort of adults on computerized measures of cognitive control, including working memory, switching, as well as risk‐taking. We also collected data on individuals with a lifetime history of depression and/or those with a relative with a mood disorder, although this was not planned at study initiation. We would not expect those with depression or a relative with a mood disorder to show this benefit, as previous literature demonstrates cognitive impairments in depression/anxiety [e.g., (Baune, Fuhr, Air, & Hering, 2014; Lee, Hermens, Porter, & Redoblado‐Hodge, 2012)] and there is little support for the stability/flexibility dimension for depression/anxiety. At the same time, there is a growing body of work demonstrating the overlap in the genes for both psychosis and depression (e.g., (Purcell et al., 2009). If there is a significant shared genetic burden, individuals who have mood phenotypes likely have underlying psychosis phenotypes, or at least, we would argue that expression of resilience might require avoiding expression of the shared genetic load. If our hypotheses are confirmed, it would provide preliminary data consistent with the view that switching may have “protective” functions and may illuminate possible prevention targets.

## 2 METHODS

### 2.1 Participants

**Figure 1:**
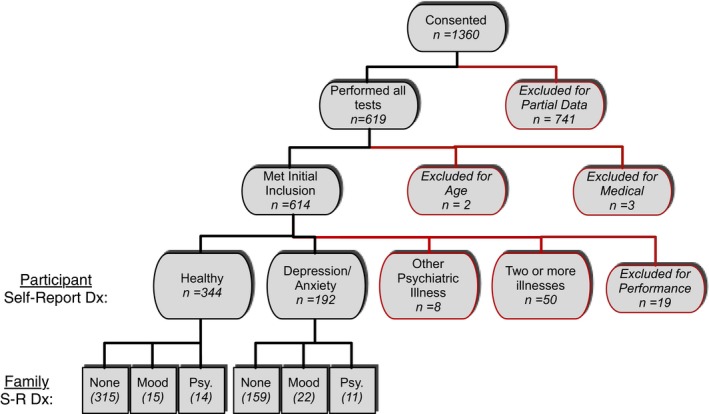
Consort Diagram: Shows the status of all participants who completed consent online. Only individuals who performed all tests were included in analysis; participants with partial data were excluded. Additional participants were excluded for the reasons identified. Number in parentheses indicates sample size. None, no family history; Mood, mood disorder (bipolar disorder or major depression); Psy, psychosis (schizophrenia, schizoaffective, bipolar w/psychotic features). S‐R, Self‐Report; Dx, Diagnosis

A total of 1,360 volunteers from the community underwent informed consent procedures online as approved by UCLA IRB. English‐speaking individuals between the ages of 18 and 65 were eligible. Advertisements specifically encouraged individuals with family histories of mood and psychotic disorders to participate. Six hundred and fourteen individuals met initial inclusion criteria (Figure 1). Participants received a $25 gift‐card as compensation after verification of identity by our study coordinator. Additional exclusion criteria were self‐reported diagnosis of autism spectrum disorder, ADHD, schizophrenia, bipolar, and/or schizoaffective disorder.

Of those who completed testing (*n* = 619), two individuals were excluded due to reporting ages out of range. One individual was excluded due to self‐report of 22q11.2 deletion syndrome in a parent. Three individuals were excluded for not providing answers about their mood and other psychiatric illness status. Fifty‐eight people were excluded due to self‐report of a diagnosis other than “depression/anxiety”. Nineteen individuals were excluded for performing at 55% accuracy or less on the lowest working memory load, demonstrating they were not adequately attending to the task. Thus, 536 participants were included in the present analysis.

### 2.2 Procedure

All testing was done online at BrainTest.org (Sabb et al., 2013). After completing online consent procedures, participants could complete the measures in any order they preferred. They were told they could take as many breaks as they wanted but were required to finish within 7 days. For all tests and surveys, a new window is opened and maximized on the participant's screen. For each test, they first read task instructions and completed practice trials to orient themselves to the proper key assignments, during which feedback was given.

### 2.3 Measures

#### 2.3.1 Medical Questionnaire

We developed a self‐report medical survey that contained 22 items that broadly covered central nervous system conditions. No answers on the medical form excluded participation; however, we excluded a number of individuals from analyses, as mentioned above (Figure 1). We additionally asked individuals to report whether they had a parent, sibling, or child with a diagnosis of bipolar disorder, schizophrenia, or schizoaffective disorder. Participants could also provide a free response about their diagnosis.

The following cognitive tests were chosen a priori to examine different putative subcomponents related to cognitive control. They were the only cognitive tests run in this cohort.

#### 2.3.2 Spatial Working Memory (SWM)

Our SWM paradigm was developed using Flash (Adobe Systems) and designed to mimic laboratory‐based versions as closely as possible. Our group has extensive experience both in the laboratory and on the Web with this task (Sabb et al., 2013). Participants performed four blocks of 16 trials. These data were collected in real time on the client machine and sent back to the server at the end of each trial block using a 128‐bit encrypted connection. Participants saw 1, 3, 5, or 7 dots presented on the screen in an abstract array for 2,000 ms. After a delay of 3,000 ms., a “probe” dot appeared for 3,000 ms. and participants pressed one of two keyboard keys to indicate whether the probe dot appeared in the same location as any of the dots previously presented. Working memory load (number of dots) was randomized across trials. Both reaction time (RT) and accuracy at each level of load were examined for construct validation, however, WM Capacity (C) was calculated using hit rate (HR), correct rejection rate (CR), and number of items (*n*) [C = *n* (HR‐CR‐1)] for each load following Cowan (Cowan, 2000), and Maximum Capacity for each individual (e.g., (Karlsgodt et al., 2009)) was used as the primary indicator in the profile analysis. Feedback was given during practice trials only.

#### 2.3.3 Task switch paradigm

**Figure 2:**
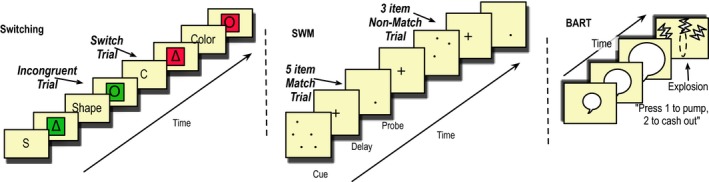
Task Schematics. Left: Task‐Switching: Cues were presented before each stimulus with either a long (1,200 ms) or short (200 ms) interstimulus interval between cue and stimulus. Participants pressed one of two keys to respond to the stimulus. Switch trials were equally split across shape/color combinations and occurred on 33% of trials. Half of the switch trials were congruent (i.e., the response indicated by the nonselected task is the same as the one indicated by the selected task) and half were incongruent.; there are four possible counterbalancing conditions for key‐category assignments. Middle: Spatial Sternberg WM: The WM set of 1,3,5, or 7 dots was presented followed by a fixed delay of 3 s. A probe dot then appeared and participants pressed a “match” or “no match” key. WM loads were counterbalanced. Right: Balloon Analogue Risk Task: A Red or Blue Balloon appears on the screen an may be incrementally inflated by button press. Each press scores a point. Another button allows the participant to “cash out” their points. If the balloon explodes, they lose their points. Red and Blue balloons have different probabilities for explosion

We used a cued task‐switching paradigm similar to that of Miyake and colleagues (Friedman et al., 2007; Miyake et al., 2004). On each trial, participants were cued with either a word or a letter indicating a stimulus dimension (e.g., “shape” or “s”) followed by a variable delay (200 or 1,200 ms.), after which they would make a forced choice response to the cued dimension of one of four stimuli (red or green, triangle or circle). A fixed poststimulus delay preceded the next trial (Figure 2).

The task consisted of 192 trials. Before the task, each participant completed 10 practice trials. The cues alternated between word and letter cues “SHAPE” and “S” or “COLOR” and “C” to avoid concerns with directly linking cue and response (Logan & Bundesen, 2003). The amount of time between cue and stimulus (CSI) was also varied; either 200 ms. (“short”) or 1,200 ms, (“long”). For the 200 ms. Cue, the interval between response and cue to next trial (RCI) was 1,600 ms., whereas for the 1,200 ms. Cue, the RCI was 600 ms. Participants saw one of four counterbalanced response mappings: 1: Left = [red OR triangle], Right = [green OR circle], 2: Left = [red OR circle], Right = [green OR triangle], 3: Left = [green OR triangle], Right = [red OR circle], 4: Left = [green OR circle], Right = [red OR triangle]. Switch trials were equally split across shape/color combinations. The main indicator, the effect of switching (or switch cost) can be examined by comparing trials on which participants switched stimulus trial‐type to ones where they repeated the same trial‐type. Although still a point of debate, short ISI trials have been frequently used to determine cost of switching (Meiran et al., 2000; Ruge, Braver, & Meiran, 2009; Vandierendonck, Liefooghe, & Verbruggen, 2010). The components of longer ISI switch costs, sometimes called “residual switch cost,” may be less clear and allow individuals time to complete task preparation (Monsell, 2003; Ruge et al., 2009); thus, we planned to use short ISI trials only for our analyses. Participants were given performance feedback during practice trials to ensure proper encoding of button responses, but were not given feedback during test trials. As with the SWM task, responses were logged on the client machine and transferred with encryption.

#### 2.3.4 Balloon Analog Risk Task (BART)

We used the BART (Lejuez et al., 2002) to investigate risk‐taking and impulse control in order to determine the specificity of our hypothesized effect of switching alongside other putative indicators of control. While not a traditional measure of cognitive control, there is evidence that BART is related to cognitive control, risk taking, and impulsivity (Bilder et al., 2004; Crow, 1997; Hanson, Thayer, & Tapert, 2014; Kóbor et al., 2015; Miller & Cohen, 2001; O'Reilly, 2006; Panwar et al., 2014; Ramey & Chrysikou, 2014; Robbins & Arnsten, 2009)). For instance, individuals who have better working memory demonstrate less impulsive and risky behavior (Khurana et al., 2015). However, one may also posit that if more risk‐taking is associated with lower (response) inhibition, it might be associated with better switching performance (e.g., (Hanson et al., 2014)). On each trial, participants are presented with a balloon in one of two colors (red/blue), and they have the option to press a button to inflate the balloon or “cash out” at any time. With each press to inflate the balloon, they earn points, unless the balloon explodes at which point they lose all their points for that particular balloon (i.e., trial). The two balloons explode at different probabilities for number of inflation presses (red = 1/32 and blue = 1/128). The objective then is to maximize points without the balloon exploding. The task has 30 trials total. Consistent with the literature, we used four indicators of performance: mean number of adjusted pumps for each color (i.e., number of pumps for trials on which there was not an explosion; 19) and number of explosions for each color.

### 2.4 Validation steps

Following our earlier work (Sabb et al., 2013), we employed an established validation approach to examine elements of our web‐based adaptations of these widely used laboratory paradigms. All our tasks are designed to be identical to laboratory‐based versions, exhibiting high face validity with well‐known and well‐validated measures. We also employ a convergent validation approach frequently used in other forms of psychological testing (McDonald, 1999; Messick, 1989).

### 2.5 Profile analysis

To maximally use the information available in this unique sample, we decided to use profile analysis to determine if—and how—our groups differ from each other across the whole range of variables of interest. Profile analysis has the advantage that it is a truly multivariate approach and its test statistics account for the autocorrelations between the different variables observed on the same participant and utilize all within and between participant information available in the sample. While other approaches to characterize groups of observations in high‐dimensional space such as multidimensional scaling or any of the types of discriminant analysis provide potentially stronger tools to characterize complex group boundaries, profile analysis provides independent tests for parallelism, level and flatness of the different groups profiles provides a close match to the questions of interest for us: Do some groups show deficits compared to others? If so, are the deficits generalized deficits across all dimensions or are they specific? Additionally, the straightforward relationship between unidimensional post hoc tests to the multivariate omnibus tests allows us to characterize the complex high dimensional results easily in a way that links directly to the underlying measures.

In order to build a cognitive response profile across our three domains to compare the relative performance of the participants on the measures of interest, we created our profile set from the primary indicator from each task as commonly used in the literature: Switch cost at the short cue–stimulus interval from Task‐Switching, Capacity from SWM; Mean Adjusted Pumps for Blue Balloons from the BART. These variables were first normed on the performance of the healthy sample, such that the participants who reported no affected relatives, and not being affected themselves have a mean score of zero and a standard deviation of one on all measures. Indicators were scored in the direction such that worse performance was equated with a larger *Z*‐score. Thus, in task‐switching, where smaller cost typically means better cognitive performance, we reverse scored it for ease of presentation. The response profiles of the different groups were then compared using a mixed model in SPSS to account for the multiple measured variables for each participant. Using age and sex as covariates did not affect the results, so we retained them in the model. The main comparison of interest is the three‐way interaction between participants two between subject factors in the model (self‐reported mental health status and the reported mental health status of the relative), and the within‐subject factor type of measure (working memory and switching, risk‐taking).

## 3 RESULTS

### 3.1 Sample characterization

The mean age of the sample was 31.8 (18–65; standard deviation [*SD*] = 9.7), and 68% were women. A little over a third (36%; 192 individuals) self‐reported a lifetime mood‐related diagnosis (major depression or bipolar disorder without psychotic features). Participants also reported about their relatives who had psychiatric diagnoses, with 7% (*n* = 37) of the respondents reporting a relative with a mood‐related diagnosis, and 5% (*n* = 25) report a relative with a psychotic disorder diagnosis (“bipolar with psychotic features”, “schizoaffective”, or “schizophrenia”). While these numbers are based on self‐report and are higher than population incidence rates (Kessler, 1994), they were not unexpected given our targeted recruitment of individuals with family histories of mood and psychotic disorders and broad inclusion/exclusion criteria for testing a community sample. Notably, our sampling strategy and recruitment led to an imbalanced sample size, with small groups of relatives with psychosis and mood disorders. As examination of subgroups, however, suggested we did not violate the assumption of equal variance, we conducted analyses on the full sample (Table 1 for age and sex for each sub group).

**Table 1:** Descriptive statistics. Provides summary measures for all groups and profile analysis measures

Participant	No DX			Depression		
Relatives	No DX *N* = 315	Mood *N* = 15	Psy *N* = 14	No DX *N* = 159	Mood *N* = 22	PSY *N* = 11
Age mean (*SD*)	31.3 (9.6)	31.3 (9.6)	34.7 (7.7)	31.2 (10.1)	34.8 (11.2)	35.2 (6.8)
Sex (% Female)	63%	73%	71%	76%	86%	45%
Primary indicators used in the Profile analysis						
TS SwitchCost (Short ISI)	124.98 (131.27)	193.36 (223.63)	51.01 (110.09)	131.45 (132.87)	142.76 (145.09)	260.864 (242.63)
WM Max Capacity	4.56 (1.46)	4.14 (1.68)	4.58 (1.57)	4.70 (1.27)	4.14 (1.35)	3.69 (1.51)
BART MAP (blue balloons)	18.20 (12.63)	21.52 (15.53)	21.74 (15.27)	18.57 (11.36)	22.98 (19.38)	21.02 (18.64)
Other dependent variables of interest						
WM Load 1 RT	850 (216)	877 (195)	949 (221)	902 (217)	898 (196)	878 (279)
WM Load 1 Acc	0.948 (0.071)	0.917 (0.097)	0.926 (0.113)	0.957 (0.069)	0.939 (0.074)	0.934 (0.108)
WM Load 3 RT	1,017 (232)	1,060 (257)	1,083 (182)	1,088 (233)	1,041 (196)	1,077(333)
WM Load 3 Acc	0.896 (0.104)	0.851 (0.172)	0.904 (0.120)	0.891 (0.114)	0.894 (0.100)	0.83 (0.123)
WM Load 5 RT	1,099 (251)	1,092 (190)	1,225 (261)	1,179 (250)	1,133 (185)	1,168 (386)
WM Load 5 Acc	0.827 (0.119)	0.777 (0.183)	0.877 (0.112)	0.818 (0.119)	0.828 (0.130)	0.757 (0.158)
WM Load7_RT	1,121 (249)	1,173 (242)	1,213 (315)	1,180 (240)	1,136 (176)	1,225 (423)
WM Load7_Acc	0.811 (0.129)	0.781 (0.147)	0.797 (0.171)	0.824 (0.107)	0.76 (0.159)	0.757 (0.081)
TS RT	847 (290)	1,015 (359)	845 (158)	893 (271)	864 (254)	1,136 (405)
TS Acc.	0.953 (0.055)	0.954 (0.037)	0.954 (0.061)	0.954 (0.058)	0.97 (0.015)	0.911 (0.121)
TS Repeat RT	846 (277)	987 (293)	879 (206)	896 (271)	851 (248)	1,070 (339)
TS Switch RT	971 (332)	1,180 (440)	930 (186)	1,027 (338)	994 (309)	1,331 (513)
BART Explosions (Red)	8.01 (3.5)	8.6 (3.3)	8.43 (2.4)	8.33 (3.3)	8.55 (3.3)	6.72 (2.2)
BART Red RT	441 (275)	472 (150)	490 (184)	430 (191)	437 (166)	692 (496)
BART MAP (red balloons)	17.57 (11.47)	16.98 (10.45)	22.41 (16.45)	18.76 (11.67)	21.33 (16.95)	20.67 (15.52)
BART Explosions (Blue)	2.18 (2.1)	1.13 (2.3)	1.36 (1.3)	2.18 (2.2)	2.32 (2.2)	2.64 (3.2)
BART RT (blue)	385 (187)	442 (164)	443 (186)	379 (168)	394 (146)	519 (302)

Acc, accuracy; BART, balloon analogue risk task; Dx, self‐reported diagnosis; ISI, interstimulus interval; Load7, working memory load 7; MAP, mean adjusted pumps; MOOD, relative with a mood disorder; PSY, relative with psychosis; RT, reaction time; TS, task‐switching; WM, working memory.

Data are mean values with standard deviations in parentheses. Five primary measures were used in the profile analysis: TS Switch Cost, WM Load7 Reaction Time, WM Load7 Accuracy, BART Mean Adjusted Pumps Red Balloons, BART Mean Adjusted Pumps Blue Balloons). Additional dependent variables are provided for more broad comparison within tasks and were examined in validation steps to ensure tasks were working as expected, but were not used in the profile analysis.

We observed overall differences in the reported patterns of illness of the relatives, based on the mood status of the participants (chi‐squared [χ^2^] (2) = 10.3, *p* < .01), Participants with mood disorders reported a significantly higher incidence of relatives with mood disorders (11%) than participants without mood disorders (4.4%, χ^2^ (1) = 9.56, *p* < .01), but not the incidence of relatives with psychosis (6% and 4% respectively, χ^2^ (1) = 1.11, *p* = .29).

**Figure 3:**
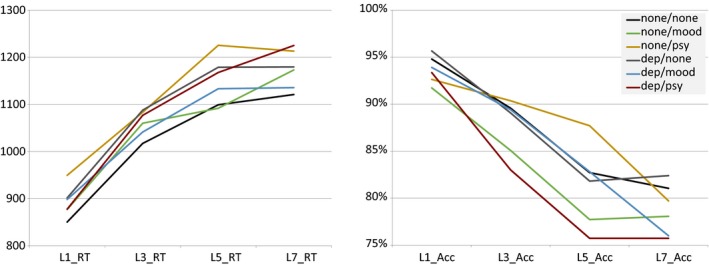
Working Memory by load. In order to help demonstrate construct validity, we show predicted patterns within task. The data show working memory decreases in performance as the levels of memory load increases for each group in reaction time (left) and accuracy (right)

**Figure 4:**
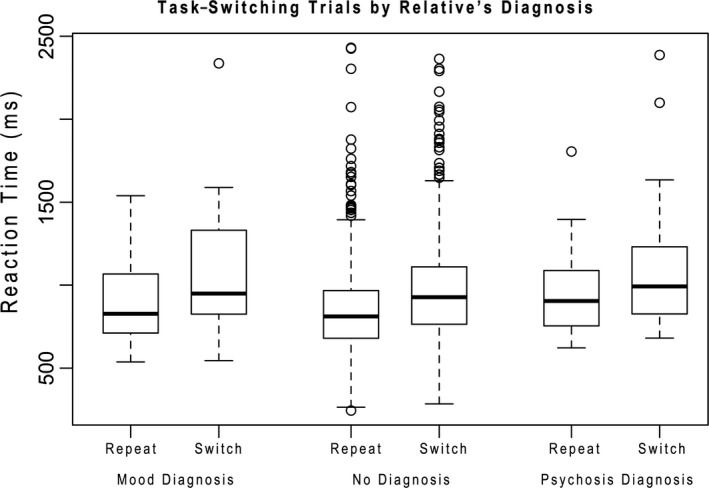
Task‐Switching repeat and switch trials by Relative's Diagnostic Group. Shows box plots of reaction time for trials had the same (repeat) and different (switch) stimulus dimensions cued on the previous trial only in participants who reported no diagnosis (self‐reported healthy). Reaction time is in milliseconds. Box plots depict median and quartiles

To ensure our tasks were performing as expected, we first examined summary statistics for our tasks and indicators (Table 1). Overall performance was consistent with what we have seen previously and are consistent with laboratory performance (Sabb et al., 2013). Accuracy decreased with increased SWM load and reaction times increased (Figure 3). Similarly, task‐switching showed expected performance characteristics, with larger switch costs for short‐duration intervals than long‐duration intervals, consistent with those seen in the literature (Meiran et al., 2000), and BART showed larger numbers of explosions for higher probability balloons (Lejuez et al., 2002) (Table 1 and Figure 4). Analysis of trial level data in the no diagnosis group only showed high reliability for task‐switching switch (alpha = 0.94) and nonswitch (alpha = 0.96) trials, working memory RT data across number of items (alpha Load 1 = 0.84, Load 3 = 0.85, Load 5 = 0.84, Load 7 = 0.83) and number of pumps in nonexploded Blue Balloon trials (alpha = 0.97), but lower reliability for accuracy data in the working memory task across number of items (alpha Load 1 = 0.40, Load 3 = 0.47, Load 5 = 0.39, Load 7 = 0.45).

### 3.2 Profile analysis

**Figure 5:**
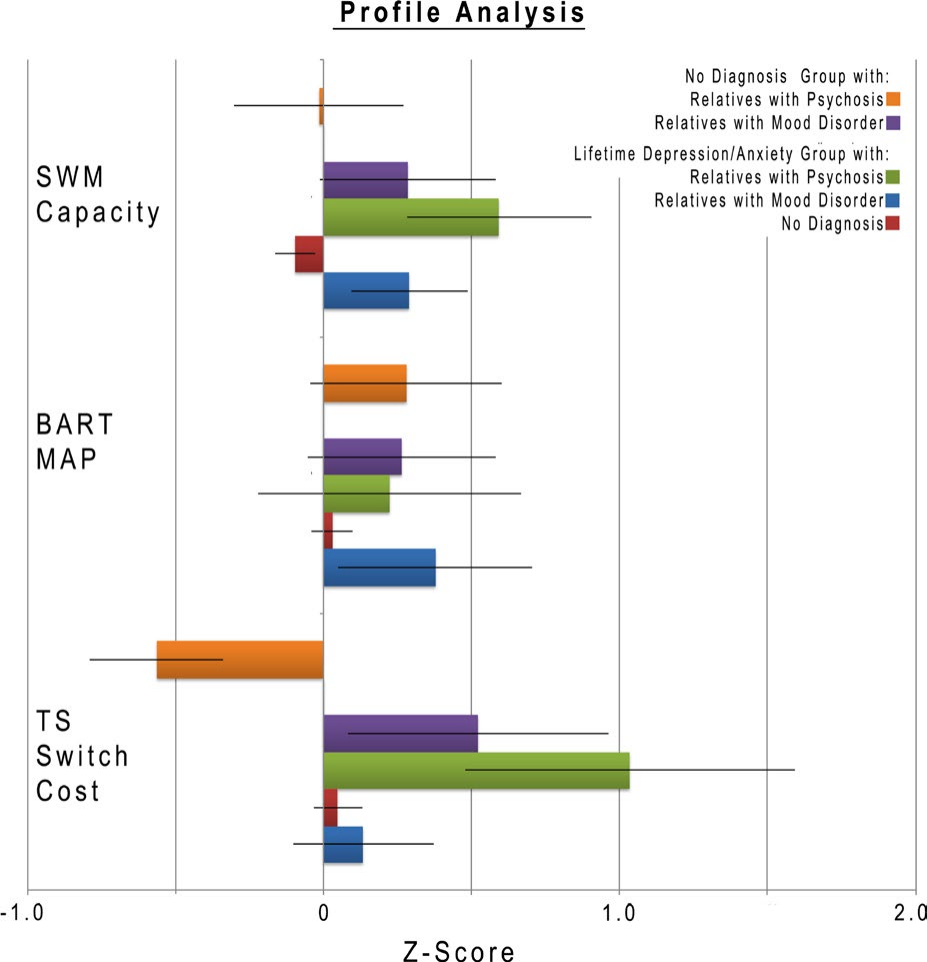
Profile Analysis. Shows *z*‐scores for 3 primary measures from the profile analysis across self‐reported diagnosis for participant and relative. All groups were normed against the no diagnosis participant group with no reported familial load as part of the profile analysis. Positive *z*‐scores represent worse performance (larger switch cost, smaller WM capacity, and greater MAP) and negative *z*‐score is better performance (smaller switch cost, larger WM capacity, and fewer MAP) for ease of presentation. Error bars depict standard error

To address our overarching hypothesis, we conducted an omnibus test followed by post hoc *t* tests to further characterize our findings (Figure 5). We found a significant three‐way interaction [*F*(4,1056) = 4.872, *p* < .01, GG *p* < .01], showing that pattern of impairment across our three main measures (Task‐Switching: Switch cost short cue–stimulus interval, SWM Capacity and BART: Mean Adjusted Pumps for Blue Balloons) is dependent on both the participants mental health status and their relatives’ mental health status. Healthy individuals who report having a relative with a psychotic disorder showed increased switching as indexed by a lower switch cost in the cued task‐switching task. This effect is specific to task‐switching, and not a general effect, as the other two tasks did not show a corresponding improvement, although we were unable to fully test discriminability between tasks (see below for further discussion; (Chapman & Chapman, 1978; Melinder, Barch, Heydebrand, & Csernansky, 2005)). This pattern is specific to healthy participants and is not seen in individuals who reported having a lifetime mood‐related diagnosis. We also found a significant difference in the measurement profile related to the relatives’ diagnosis (Dx) *F*(4,1056) = 2.77, *p* = .03, GG *p* = .03]. To further elaborate on the structure of these interactions, we conducted post hoc analyses on the two‐way interactions within each of the participant groups that make up this three‐way interaction.

For participants that self‐report as healthy, we observed a significant two‐way interaction between the type of measure and mental health status of the relative [*F*(4,678) = 2.81, *p* = .02, GG *p* = .02]. This significant two‐way interaction can be decomposed into three sets of main effects. For short switch cost (Costsh), there is a significant main effect of the relatives’ reported mental health status (*F*(2,339) = 4.65, *p* = .01) with the participants with relatives with a psychotic disorder (estimated mean = −0.64, *SE* = 0.27) showing significantly better performance than participants with a relative with a mood disorder (estimated mean = 0.51, *SE* = .26, *p* < .01), and participants within the no‐diagnosis group (mean = 0, *SE* = .06, *p* = .02). The no‐diagnosis group is not significantly different from the group with a relative with a mood disorder (*p* = .06). There are no significant effects of the relatives’ mental health status on WM capacity (*F*(2,339) = 0.55, *p* = .57) or BART (Mean‐Adjusted Pumps Blue Balloons: *F*(2,339) = 1.09, *p* = .34).

For participants that self‐report as having a history of depression/anxiety, there is a significant interaction between the type of the measure and the mental health status of the relative (*F*(4, 374) = 4.32, *p* < .01, GG *p* < .01). This significant two‐way interaction can be decomposed into three sets of main effects. For task switch cost (Costsh), there is a significant main effect of the relatives mental health status (*F*(2,187) = 4.147, *p* = .017). The participants with relatives they report to have psychosis (estimated mean = 1.01, *SE* = .32) perform significantly worse than participants who report relatives with mood disorder (estimated mean = 0.03, *SE* = .22, *p* = .01) and those with relatives with no diagnosis (estimated mean = 0.06, *SE* = .08, *p* < .01). The participants whose relatives have no diagnosis are not different from those with mood disorder relatives (*p* = .89).

For SWM Capacity, there is no significant effect of the relatives mental health status (*F*(2,187) = 4.31, *p* = .01) in this group. The participants with relatives they report to have psychosis (estimated mean = −0.81, *SE* = 0.27) perform significantly worse than the participants with relatives with no diagnosis (estimated mean = 0.10, *SE* = 0.10) ( *p* = .02), but not significantly different from participants with relatives reported to have a mood disorder (estimated mean = −0.29, *SE* = 1.91, *p* = .39). The participants whose relatives have no diagnosis perform not significantly different from those with mood disorder relatives (*p* = .06). There is no significant effect on mean‐adjusted pumps in the BART (Map Blue *F*(2,187) = 1.27, *p* = .28). All analyses control for age and sex effects and the reported estimated means are adjusted for age and sex.

In order to examine whether our imbalanced design significantly altered the results, through overweighing the larger subsamples in the estimation of the pooled within subject variability, we conducted a secondary analysis using an age‐ and sex‐matched subsample (*n* = 14). Using this matched sample, the three‐way interaction is reduced to a trend level (*F*(4,672) = 2.08, *p* = .081, Greenhouse‐Geiser [GG] *p* = .081) most likely due to reduced power from the small sample size. Importantly, the main effect that underlies the three‐way interaction in the larger sample still shows a substantial effect size (d = .67) but is no longer significant (Cost‐switching for the no diagnosis group with relatives who report psychosis = −0.18 (1.00), Cost‐switching for the no‐diagnosis group and relatives who report a mood diagnosis = 1.03 (1.85), *F*(1,13) = 1.52, *p* = .24). None of the other cognitive measures showed significant differences or comparable effect sizes to the main finding: BART Map‐Blue: *F*(1,13) = .14, *p* = .71, d = .19, SWM Capacity: *F*(1,13) = .11, *p* = .74, d = .19).

## 4 DISCUSSION

Consistent with our overarching hypothesis, we found a significant interaction whereby healthy individuals at familial risk for psychosis performed significantly better on a cued task‐switching task, relative to all other groups examined. Importantly, our finding of better switching performance is not consistent with a generalized deficit model of risk for psychosis. Individuals with a psychotic relative who self‐report being healthy showed better performance than individuals with no reported family history. This was a selective benefit seen only in task‐switching and not in working memory or risk‐taking tasks, which have been previously linked to cognitive control. Furthermore, this effect was only seen in those individuals who were healthy, and not those who reported lifetime occurrence of depression or anxiety, who showed more impaired switching than all other groups. The inclusion of this latter group was not part of the original study design; however, a significant number of people reported lifetime occurrence of depression or anxiety and still completed all measures. While preliminary, these results are suggestive of a potentially adaptive (or compensatory) processes in healthy individuals who are at higher risk for psychosis due to familial genetic load. This may provide a useful framework for examining the neurobiological underpinnings of psychotic disorders and help reduce stigma associated with these brain illnesses.

Previous studies examining relatives of those with psychosis frequently find that relatives show an intermediate phenotype, where their performance is midway between healthy and unwell probands. A rigorous meta‐analysis of cognitive deficits in unaffected relatives of those with schizophrenia by Snitz and colleagues (Snitz et al., 2006) finds small to medium effect sizes across a wide variety of cognitive indicators including deficits in working memory, WCST, and the AX‐version of the CPT (AX‐CPT, e.g., (Braver & Barch, 2002)). For instance, Macdonald et al. (2003) found that relatives had deficits in context processing, using the AX‐CPT. This led to better performance by relatives on trials where the cue was incorrect. This specific pattern of errors in relatives, however, suggested they did not process the cue–stimulus in the same way as those without presumed genetic risk, thus perhaps less likely to be considered a true cognitive benefit.

Other indicators examined in the Snitz et al. (2006) analysis, however, did not show superior performance among unaffected relatives, although several individual studies presumably found negative effect sizes (i.e., better performance) in prosaccade and Stroop tasks given confidence intervals that extend below zero. Importantly, the tasks most frequently examined (WCST and TRAILS‐B—which show moderate effect sizes) may not be optimal for examining switching. These traditional neuropsychological measures assay a range of cognitive functions including working memory and typically show medium to large deficits in relatives (Snitz et al., 2006; Szöke et al., 2008). Pointedly, a meta‐analysis by Li (2004) also cautions against simplification of WCST results in schizophrenia as errors related to a switching component, finding evidence for relatively similar numbers of perseverative and nonperseverative errors, suggesting it may not be measuring the ability to switch.

The cognitive and neural underpinnings of task‐switching measures are still active areas of research (Kenner et al., 2010; Ruge et al., 2005), but are designed to selectively manipulate the switching process experimentally in order to identify the key mechanism in the switch cost. There remains debate over whether it is related to an active switching process, inhibition, or an effect of stimulus priming [see Meiran (2000a) for review]. While we cannot determine whether our effect is due to an active process or inhibition or priming, it does putatively demonstrate less overlap with other constructs than WCST/CANTAB as evidenced by neuroimaging studies (Ravizza, Moua, Long, & Carter, 2010). It is not clear how our findings of benefits in switching in unaffected relatives of those with psychosis may interact, but further work in this area is warranted.

We found a significant difference in task‐switching that was not observed in other measures. This result, however, could be complicated by a difference in discriminatory power in each of these tasks (Chapman & Chapman, 1978; Melinder et al., 2005). While we have some evidence to suggest the internal consistency (Cronbach's alpha) and variance across groups could not completely account for these findings, we were not in a position to conduct a true discriminatory analysis, and thus more work needs to be done to examine whether the task‐switching task is merely a more discriminating measure.

This preliminary work investigates an important aspect of behavioral health related to risk for psychopathology, but there are a number of limitations that need to be addressed with further investigation. We examined individuals who self‐report being healthy and those who self‐report having had a lifetime diagnosis of depression or anxiety. Crucially, this work relies on self‐report of participants’ health status and their subjective report of the health status of their relatives rather than objective testing by a clinician, which is needed to replicate these findings. Relatedly, this cohort was collected and run entirely over the Web, and while numerous studies have showed the validity of online experiments (Germine et al., 2012; Gosling, Vazire, Srivastava, & John, 2004; Haworth et al., 2007; Krantz & Dalal, 2000), further validation using rigorous laboratory‐based clinical measures would be fruitful. While our results using these tasks are consistent with what is seen in the laboratory, and our recruitment methods were similar to many laboratory‐based studies, further validation will be helpful in assessing the generalizability of these results. While not a true epidemiological sampling approach, our approach to recruitment helps provide insight into the population of individuals who perform web‐based experiments and minimizes false‐responding as there few exclusions and all were allowed to perform the measures (i.e., no response was conditional for full participation).

Our tasks did not control for stimulus modality, and while we would not have expected a stimulus‐modality bias in these data, further work would need to investigate this potential confound. While the BART does not help provide insight into the specific cognitive process underpinning our finding, it does help provide evidence at the latent construct level that cognitive control is not broadly implicated. Follow‐up work with a larger battery of more fine‐grained tasks will be needed to further characterize these effects, however, the BART provides preliminary evidence that risk‐taking or impulsivity may not be involved as a mitigating factor. We also note that our implementation of the task‐switching task involved trials of equal length, specifically the interval between the response and the next trial differed. While previous research has showed that intervals greater than 550–650 ms have little effect of the switch cost (Meiran, 2000b; Monsell, 2003), this could be directly assessed with future work.

We also further highlight that our broad sampling strategy led to imbalanced sample sizes in each group, although in examining a more balanced group, we found similar variance in the control group, suggesting the imbalanced design did not significantly alter the findings. We ran the analyses with and without covarying for age and sex, which did not alter the main finding, yet given our small unbalanced sample sizes, it remains potentially problematic and requires independent validation preferably by recruiting matched samples. We also cannot discern how much of this “benefit” may be due to genetic predisposition vs. learned or environmental factors (such as education). These findings require validation with well‐characterized laboratory‐based cohorts, preferably with direct testing of multiple family members, which could uncover the contribution of genetic/environmental influences. While here we focus on the potential benefits of switching, the balance between active maintenance and flexibility is essential, and there is some evidence these constructs lie on a continuum (Bilder et al., 2004; Durstewitz et al., 1999; Miller & Cohen, 2001). We also did not find a deficit in WM capacity for our no‐diagnosis group with familial risk for psychosis, which was surprising. There is strong support for nonill relatives of those with psychosis to show WM deficits; however, as seen in Snitz et al. (Snitz et al., 2006), several studies report finding negligible effect sizes in Spatial WM, and there could be a “file drawer” problem for published reports with negative findings. Further work is needed to examine the cause of our WM finding. Finally, we also have very limited information reported by participants about their current medications. While participants were asked to exclude themselves if they were taking any medication known to affect the brain including antidepressants, it was not possible for us to verify that in the scope of this study.

## 5 CONCLUSIONS

While preliminary, this novel finding shows that healthy individuals with a familial risk for developing psychosis have enhanced switching as compared to healthy individuals without familial risk and individuals with a personal or family history of depression or anxiety. This may provide a framework for validation and replication that incorporates familial risk from putative genetic load, behavioral performance, and outcome. Healthy individuals may or may not have genetic risk for mood or psychotic disorders, which may include both disease‐selective and nonselective genetic contributions. We have examined behavioral correlates of these overlapping features, finding a nonlinear relationship between behavioral variables and clinical outcome. Switching, but not working memory or risk‐taking, was able to dissociate individuals who have not succumbed to their familial risk. Noticeably absent are direct brain and symptom measures, which require laboratory visits for adequate measurement, and represent a future direction of this research. This potentially adaptive or compensatory mechanism may provide a novel approach for examining the underlying neural and genetic mechanisms associated with these brain illnesses and may further reduce stigma by demonstrating positive cognitive adaptations associated with vulnerable phenotypes that may identify relative strengths that can be leveraged in preventive interventions.
